# Quality of life among total laryngectomized patients undergoing speech rehabilitation: correlation between several instruments

**DOI:** 10.6061/clinics/2020/e2035

**Published:** 2020-11-02

**Authors:** Ana Carolina Soares Raquel, Elaine Pires Buzaneli, Hevely Saray Lima Silveira, Marcia Simões-Zenari, Marco Aurélio Valmondes Kulcsar, Luiz Paulo Kowalski, Kátia Nemr

**Affiliations:** I Programa de pos-graduacao em Ciencias da reabilitacao, Faculdade de Medicina (FMUSP), Universidade de Sao Paulo, Sao Paulo, SP, BR; II Departamento de Fisioterapia, Fonoaudiologia e Terapia Ocupacional, Faculdade de Medicina (FMUSP), Universidade de Sao Paulo, Sao Paulo, SP, BR; III Instituto do Cancer do Estado de Sao Paulo (ICESP), Sao Paulo, SP, BR; IV Departamento de Cirurgia - Cirurgia de Cabeça e Pescoço, Faculdade de Medicina (FMUSP), Universidade de Sao Paulo, Sao Paulo, SP, BR

**Keywords:** Quality of Life, Laryngectomy, Alaryngeal, Speech, Head and Neck Neoplasms

## Abstract

**OBJECTIVE:**

The aim of this study was to correlate several instruments currently used for the assessment of the quality of life of patients who underwent total laryngectomy and speech rehabilitation.

**METHODS:**

A cross-sectional, observational study was conducted with 38 patients after total laryngectomy and speech therapy aiming to develop oesophageal speech. The patients were divided into the following two groups (19 participants each): speakers and non-speakers. The quality of life instruments used were as follows: visual analogue scale (VAS); Voice Handicap Index (VHI); Voice-Related Quality of Life (V-RQOL); Functional Assessment of Cancer Therapy - Head & Neck (FACT-H&N); European Organisation for Research and Treatment of Cancer Core Quality of Life Questionnaire (EORTC QLQ-C30); European Organization for Research and Treatment of Cancer Quality of Life Questionnaire-Head and Neck (EORTC QLQ-H&N35); and University of Washington Quality of Life (UW-QOL).

**RESULTS:**

The V-RQOL global health domain exhibited a strong correlation with the VHI. The EORTC QLQ-C30 exhibited a moderate to strong correlation with the EORTC QLQ-H&N35 functional domain in both groups. The EORTC QLQ-C30 functional domain exhibited a strong to moderate correlation with all other instruments in both groups. The UW-QOL exhibited a moderate to strong correlation with the VHI and EORTC QLQ-C30 in both groups.

**CONCLUSION:**

The EORTC QLQ-C30, EORTC QLQ-H&N35 and UW-QOL were the instruments that most correlated with the remaining instruments, indicating that any of the three can be used to assess the quality of life of the target population regardless of oesophageal voice development.

## INTRODUCTION

Total laryngectomy results in irreversible loss of laryngeal voice and other changes with a remarkable impact on quality of life (1-4).

In developing countries, the access to tracheoesophageal prosthesis rehabilitation may be difficult, and oesophageal voice rehabilitation may be the only available method for a significant number of patients (5-7). The wide variation in the success rate described in the literature might be due to several clinical, anatomical, physiological, cognitive or psychological/emotional factors (1,5,8).

The proficiency of oesophageal speech may impact patients’ quality of life, and this impact may differentiate those who have rehabilitated themselves from those who have not yet rehabilitated themselves. The use of tools for quality of life assessment should be part of the post-treatment evaluation and can be applied to follow-up the rehabilitation process, providing guidance for more effective planning by the multiprofessional team. Studies investigating quality of life after total laryngectomy have used several instruments. However, most of these studies use a single questionnaire, or they apply one specific voice and one specific cancer to assess different forms of rehabilitation when more than one are associated (9,10-16). Previous studies have compared different instruments for monitoring quality of life after cancer treatment but without detailing the variable of phonatoric rehabilitation (17-19). The Voice Handicap Index (VHI) (20) and Voice-Related Quality of Life (V-RQOL) (21) are among the quality of life instruments most widely used by voice specialists.

These instruments have been applied to dysphonic individuals by several aetiologies, including the population of total laryngectomized individuals in some studies. When applied to total laryngectomized individuals, these instruments are associated with a more specific assessment protocol for laryngeal cancer most of the time (22). A previous study has evaluated the quality of life using a general SF-36 instrument associated with a specific voice (V-RQOL) in heterogeneous samples, including dysphonia caused by laryngeal cancer or not caused by laryngeal cancer (23). Other studies have applied only specific voice protocols (V-RQOL and/or VHI) to investigate the vocal disadvantage, quality of life and voice as well as how these individuals deal with vocal impairment resulting from laryngectomies (12,14,24,25). The Coping Strategies in Dysphonia Protocol (PEED-27) has also been utilized to investigate strategies used after total laryngectomy (24).

Specific instruments for head and neck cancer include the University of Washington Quality of Life (UW-QOL) (26), Functional Assessment of Cancer Therapy - Head & Neck (FACT-H&N) scale (27) and the European Organisation for Research and Treatment of Cancer Core Quality of Life Questionnaire (EORTC QLQ-C30 and its specific H&N35) (28,29). EORTC QLQ-C30 and EORTC H&N35 have been applied in a study on the quality of life of laryngeal cancer patients treated with radiotherapy (30).

Some studies have applied UW-QOL to investigate individuals who have undergone total laryngectomy (19,16), to compare quality of life between a group of total laryngectomized individuals and a group of individuals who underwent organ preservation treatment (31) or for the comparison among tracheoesophageal prosthesis, oesophageal voice or electronic larynx (16).

Quality of life investigations by means of specific protocols related to head and neck cancer in which voice and communication may be inserted or associated with a specific voice protocol have also been described in the literature. EORTC QLQ-C30, EORTC QLQ-H&N35 and VHI instruments have been associated with compare quality of life in individuals rehabilitated by tracheoesophageal prosthesis, oesophageal voice and electronic larynx with better quality of life observed in individuals with oesophageal voice (15). Similarly, other researchers have compared a group of individuals rehabilitated with tracheoesophageal prosthesis, oesophageal voice and electronic larynx to a group of individuals without alarming voice. These researchers reported higher scores in the functional scales of these instruments for oesophageal speakers, and they observed the greatest negative impact in the group without vocal rehabilitation (32).

When applying UW-QOL, Souza et al. (16) observed that those with tracheoesophageal prosthesis present a better quality of life compared to those who underwent other methods. Although most evaluated their quality of life in a positive way, the authors emphasized that the absence of vocal emission is the only variable associated with a lower quality of life.

In addition, a self-report visual analogue scale (VAS) is used in several fields to measure the perception of individuals about certain events, such as pain and voice quality (33). The VAS is regarded as reliable and easy to apply (34). Some experts consider this scale for laryngectomized people, but the question involved depends on the type of question it refers to in its application. However, there is no evidence that this instrument is sufficient to assess the quality of life of total laryngectomized people because no studies have incorporated a question intended to investigate the quality of life in such cases. Kucuk et al. (35) investigated the expectations of individuals receiving treatment for laryngeal squamous cell carcinoma by applying the VAS before and after treatment, and to investigate quality of life, they used EORTC QLQ-C30 and EORTC QLQ-H&N35 instruments three months after completion of treatment. Pellicani et al. (36) analysed tracheoesophageal vocal resistance in laryngectomized patients, including one comparison of the self-assessment of signs and symptoms of vocal fatigue before and after the vocal resistance test, using the VAS, but they found no significant changes.

The instruments may converge on the questions, and the possible types of answers may vary between them. In addition, there is a scarcity in the literature regarding the evaluation of the quality of life, especially in groups without a voice. We did not find surveys comparing all of these instruments in a laryngectomized population with and without an oesophageal voice to determine if there is a correlation between them and which one could meet the needs of these individuals in relation to the evaluation of their quality of life regardless of alaryngeal voice. The use of the same evaluation instruments favours the comparison of results between different services, and this knowledge can help communication between specialists. Additionally, these findings may allow epidemiological analyses relevant to public policies aimed at improving the quality of care and the development of research seeking scientific evidence. Considering that Brazil and other developing countries still have a large number of laryngectomized people dependent on oesophageal voice as an alternative for alaryngeal communication, investigation of the potential correlations among these instruments is of great value to specialists.

## METHODS

The present cross-sectional, observational study was conducted with laryngeal cancer patients who underwent total laryngectomy or total laryngectomy with partial pharyngectomy at the Instituto do Cancer do Estado de Sao Paulo and Hospital das Clinicas, School of Medicine, University of São Paulo from 2016 to 2018.

This study was approved by Ethics Committees of the institutions (Parecer: 2.229.237), and informed consent was obtained from all participants included in the study.

### Participants

The sample consisted of patients at two institutions and who underwent speech therapy by different professionals. The therapy was based on the development of the oesophageal voice by means of air swallowing methods (37), air injection (38) and air aspiration (39), and the researcher did not participate in the sessions. Contact with patients only occurred at the time of the invitation, evaluation of inclusion criteria of individuals and application of the instruments. The participants' speech was always evaluated by the researcher and by one of the speech therapists who assisted the patient to classify by consensus the degree of oesophageal voice acquisition using the Wepman scale for classification of speakers (SG) and non-speakers (NSG) (40).

To guarantee that the level of education did not interfere with the responses, we applied the Mini Mental State Examination (MMSE) and excluded participants who obtained a final score below the cut-off value. Only individuals with associated conditions impairing comprehension, those unable to answer the instruments and those who scored below the minimum on the MMSE were excluded (minimum score less than or equal to 24 points; in the case of less than 4 years of schooling, the cut-off value changes to 17) (41).

Calculation of the sample size was performed to identify the number of patients necessary for the effectiveness of the study, and the minimum number of participants for both groups (SG and NSG) was determined to be 19. All patients undergoing speech rehabilitation at the participating institutions were included regardless of length of speech therapy or degree of voice proficiency until the sample number in each group was reached. The parameters reported by Davatz (42) were used for this calculation. For the calculation, a quarter of the amplitude and the difference between the medians were listed to replace the standard deviation. The level of significance adopted was 5%, and the test power was 80%, which allowed the approximate number of individuals in each group to be determined.

Regarding characterization of the 38 participants, ex-smokers, ex-drinkers and individuals who had undergone total laryngectomy with neck dissection and radiotherapy predominated (Table 1).

The mean age of the SG group was 59.6 years (range of 30 to 68 years), and the mean age of the NSG group was 62 years (range of 47 to 77 years). The median time since surgery was 20.6 months for the SG group (range of 7 to 103.5 months) and 15.2 months for the NSG group (range of 2.6 to 32.1 months). The median number of speech therapy sessions was 17 for the SG group (range of 6 to 55 sessions) and 11 for the NSG group (range, 3 to 31 sessions) (Table 2). The mean MMSE score was 26.5 for the SG group (range of 19 to 30) and 24.5 for the NSG group (range of 17 to 30) (*p*=0.222). A total of 20 patients (52.6%) had attended more than four years of formal schooling, and 18 patients (47.3%) had attended up to four years of formal schooling. There were no illiterate patients included in the study.

### Procedures

Before application of the instruments, the investigator assessed the level of oesophageal voice proficiency of each patient in a silent room after the following vocal tasks: a) sustained emission of vowels /a/ and /é/ with the usual intensity and frequency; b) sentences from the Consensus Auditory-Perceptual Evaluation of Voice (CAPE-V) protocol translated into Brazilian Portuguese (43); c) counting numbers 1 to 10 with the usual intensity and frequency; d) months of the year; and e) spontaneous speech. Speech was classified following the Wepman scale (40), which comprises three variables (degree, production type and speaking ability) and seven levels. Patients classified as levels 1 to 3 exhibit automatic oesophageal sound production or produce sentences or words via voluntary and continuous voice production. Patients classified as levels 4 to 7 exhibit voluntary oesophageal sound in most monosyllabic emissions (sometimes voluntary or involuntary), no production of words or full absence of sound. Based on this classification, the patients were divided into two groups as follows: speakers (SG), levels 1 to 3; and non-speakers (NSG), levels 4 to 7.

**Figure 1:**
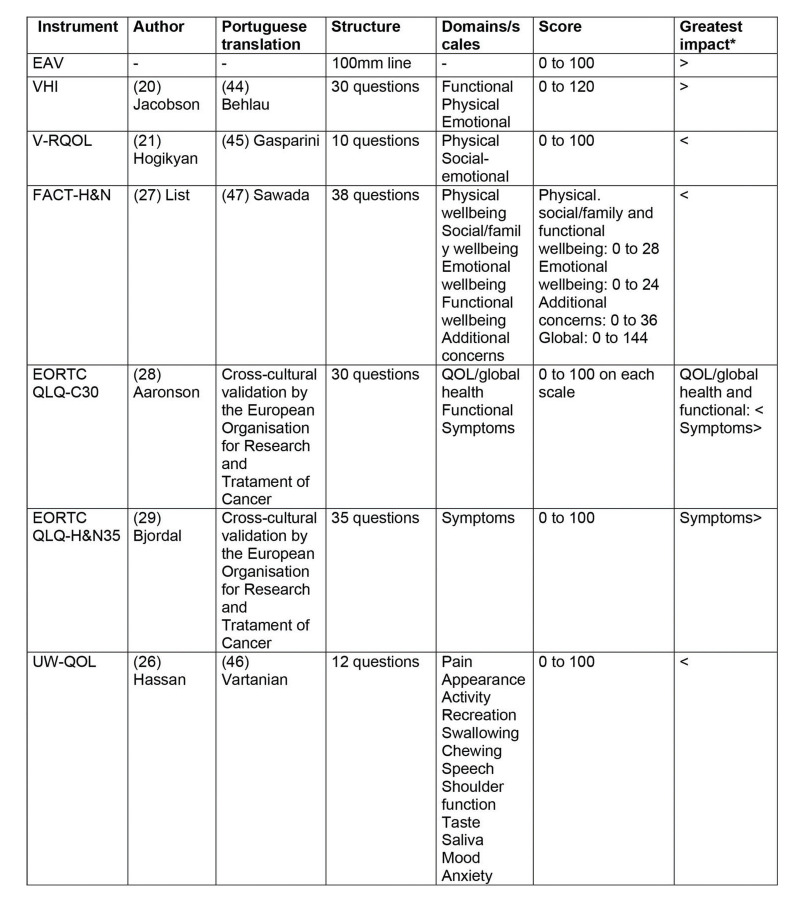
Synthesis of the applied instruments.

Seven instruments for the quality of life assessment were applied all on the same day, in the same order and by the same investigator. The main characteristics of each instrument are described in Figure 1 (20,21,26,28,29,44,45-47).

### Data analysis

Data analysis was performed by means of descriptive statistics, percentages for qualitative variables and measures of central tendency and dispersion for quantitative variables. The Shapiro-Wilk test was used to investigate the adherence of the instruments’ scores to the normal distribution. Spearman’s correlation analysis among instruments considered only the global and subdomain scores for both groups. Data analysis was performed using R-Project version 3.3.3 and Statistical Package for the Social Sciences (SPSS) version 17.0. The significance level was set at 5% in all analyses.

## RESULTS

**Figure 2:**
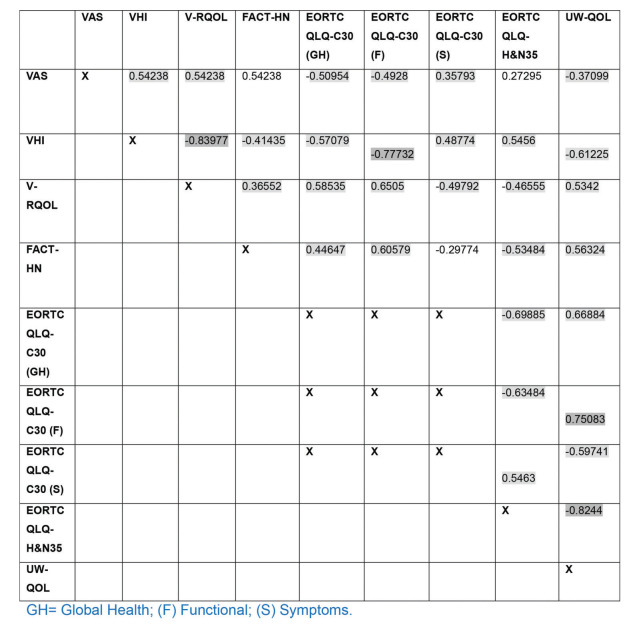
Correlation matrix among the results of the different instruments for quality of life evaluation in the total score.

In the total scores of the instruments, the VHI correlated strongly with the V-RQOL (*p*<0.001) and the EORTC QLQ-C30 functional domain (*p*<0.001). The UW-QOL instrument also strongly correlated with the EORTC QLQ-C30 functional domain (*p*<0.001) and with its specific EORTC QLQ-H&N35 (*p*<0.001). Most of the instruments exhibited moderate correlation (Figure 2).

**Figure 3:**
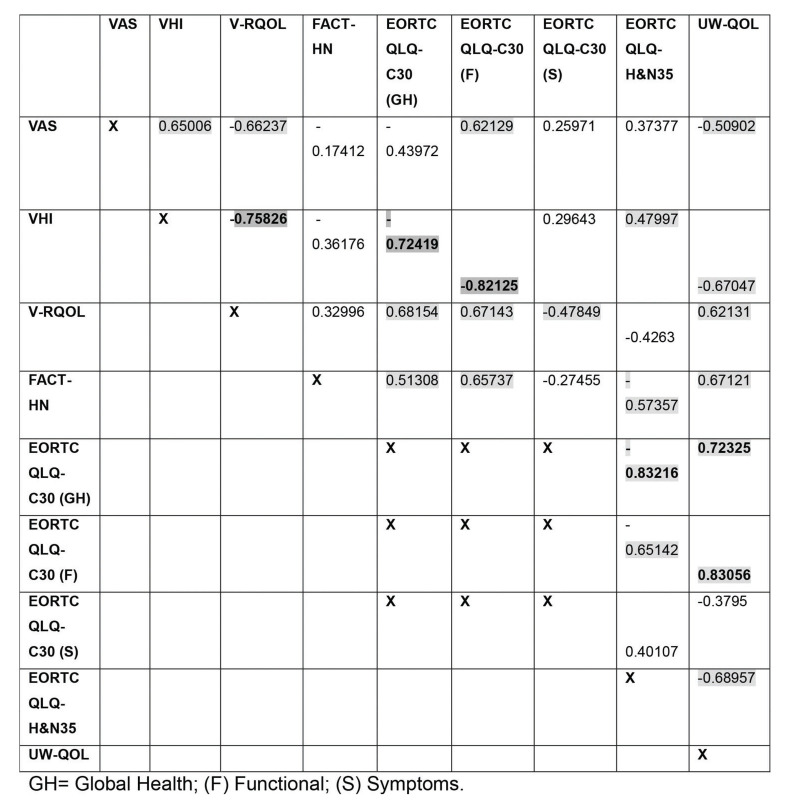
Correlation matrix among the results of the different instruments for quality of life evaluation in the SG group.

A moderate correlation was found between the VAS and all other instruments in both groups. In the SG group, a correlation was found between the VAS and the VHI (*p*=0.0026), V-RQOL (*p*=0.0002), EORTC QLQ-C30 functional domain (*p*=0.0045) and UW-QOL (*p*=0.0260) (Figure 3).

**Figure 4:**
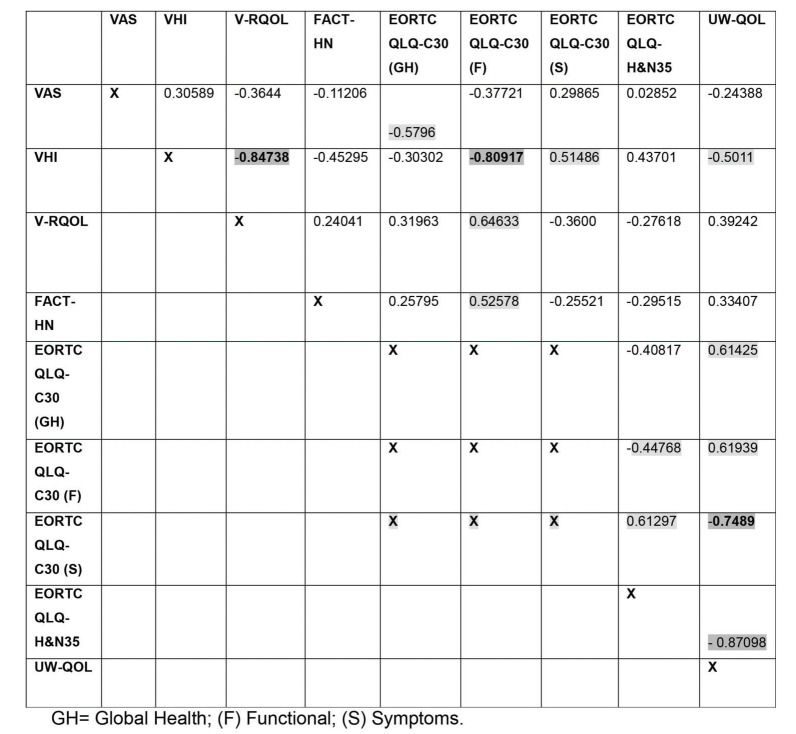
Correlation matrix among the results of the different instruments for quality of life evaluation in the NSG group.

In the NSG group, a correlation was found between the VAS and the EORTC QLQ-C30 global health status/quality of life scale (*p*=0.0093) (Figure 4).

The VHI exhibited a strong correlation with the V-RQOL global score and the EORTC QLQ-C30 functional domain (*p*=0.0002 and *p*<0.001) and a moderate correlation with the UW-QOL in both groups (*p*=0.0017 and *p*=0.0288) (Figure 3 and 4). In the SG group, the VHI exhibited a moderate correlation with the VAS (*p*=0.0026) and the EORTC QLQ-H&N35 (*p*=0.0376) and a strong correlation with the EORTC QLQ-C30 global health status/quality of life scale (*p*<0.001) (Figure 3). In the NSG group, the VHI exhibited a moderate correlation with the EORTC QLQ-C30 (*p*=0.0241) symptom domain (Figure 4).

In the SG group, the V-RQOL exhibited a moderate correlation with the EORTC QLQ-C30 global health status/quality of life scale (*p*=0.013) and symptom scale (*p*=0.0382) and with the UW-QOL (*p*=0.0045) (Table 3). In both groups, the V-RQOL exhibited a moderate correlation with the EORTC QLQ-C30 functional domain (*p*=0.0016 and *p*=0.0028) (Figures 3 and 4).

The FACT-H&N exhibited a moderate correlation with the EORTC QLQ-C30 functional domain in both groups (*p*=0.0022 and *p*=0.0208) (Figure 3 and 4). For the SG group, the FACT-H&N exhibited a moderate correlation with the EORTC QLQ-C30 global health status/quality of life scale (*p*=0.0247), EORTC QLQ-H&N35 (*p*=0.0102) and UW-QOL (*p*=0.0017) (Figure 3).

The EORTC QLQ-C30 global health status/quality of life scale exhibited a strong correlation with the EORTC QLQ-H&N35 (*p*<0.001) and UW-QOL (*p*=0.0051) in the SG group and a moderate correlation with the UW-QOL (*p*=0.0051) in the NSG group. The EORTC QLQ-C30 functional domain exhibited a moderate correlation with the EORTC QLQ-H&N35 in both groups (*p*=0.0025 and *p*=0.0500), a strong correlation with the UW-QOL in the SG group (*p*<0.001) and a moderate correlation with the UW-QOL in the NSG group (*p*=0.0047). The EORTC QLQ-C30 symptom domain exhibited a moderate correlation with the EORTC QLQ-H&N35 (*p*=0.0053) and a strong correlation with the UW-QOL in the NSG group (*p*=0.0002) (Figures 3 and 4).

The EORTC QLQ-H&N35 exhibited a correlation with the UW-QOL, which was moderate for the SG group (*p*=0.0011) and strong for the NSG group (*p*<0.001) (Figures 3 and 4).

## DISCUSSION

Several studies have categorized patients who underwent total laryngectomy into speakers and non-speakers according to the scale of Wepman (40,48,49,50). In this study, we applied different instruments to evaluate patients’ quality of life after laryngectomy according to their ability to speak or not using oesophageal speech. Although 18 participants had attended four years of formal schooling or less, resulting in a reduction in the mean global score, they did not exhibit any cognitive impairment hindering them from answering the instruments. In addition, this finding suggests that educational level does not interfere with the acquisition of oesophageal speech in the studied patients. However, several publications have shown a relationship between educational level and oesophageal speech proficiency, indicating that the former might interfere with rehabilitation (5,47). Some authors have reported that understanding the mechanism of production is a prognostic factor for oesophageal speech acquisition and that patients need to be in adequate cognitive conditions to assimilate reformulation of the mechanism of production of a new type of speech (5).

The groups exhibited similarities in sociodemographic and clinical factors. Almost all of the patients in both groups were male, in agreement with previous reports (16,32,51,52). The fact that the rate of radiotherapy was similar between the groups indicates that this factor did not interfere with the acquisition of oesophageal voice in the investigated sample. The mean age was 59.6 years for the SG group and 62 years for the NSG group, in agreement with the mean age reported in the literature for patients who underwent total laryngectomy (approximately 60 years old) (16,32).

Some studies have found no relationship among voice, speech proficiency and radiotherapy (48,53). However, other studies have reported that the voice quality might become impaired after radiotherapy with perceptible worsening after total laryngectomy (54,55).

Almost all the participants reported being both smokers and drinkers at the time of diagnosis. Previous studies describe smoking as the main cause of laryngeal tumours (56), with the risk being potentiated when smoking is combined with alcohol consumption.

The mean time since surgery was 27.6 months for the SG group and 14.5 months for the NSG group. Several studies have reported that longer lengths of time after surgery result in better quality of life after a total laryngectomy (11,14,57).

The mean number of speech therapy sessions was 25.7 for the SG group and 14.5 for the NSG group. According to the literature, longer durations of speech therapy result in better oesophageal voice proficiency and quality of life. One study concluded that the quality of life of laryngectomized patients can be improved via voice rehabilitation (13). In contrast, Souza et al. (16) observed through the UW-QOL questionnaire that the absence of vocal emission is the only variable associated with a lower quality of life.

Although we initially assumed that there would be some correlation among the questionnaires due to the similarity of some items, we did not know how the instruments would behave in both groups with and without an alaryngeal voice. The correlations between the VAS and specific voice (VHI and V-RQOL) and between head and neck (EORTC QLQ-C30 and UW-QOL) quality of life instruments in the SG group suggest that the VAS can be used with speakers because they experience a new type of speech and its limitations, which may affect the functional domain of quality of life. Another study also found a correlation between the VAS and V-RQOL (58). The moderate correlation between the VAS and EORTC QLQ-C30 global quality of life scale in the NSG group suggests that the two scales do not need to be applied for the same purpose. Future studies with larger samples should better analyse this correlation among non-speakers and in regard to other methods for voice rehabilitation after total laryngectomy.

The highly significant and inverse correlation found between the V-RQOL and VHI suggests that both can be used with this population of patients. Although the objective of this study was not to detail the subscales, the SG group and the NSG group exhibited a strong and a moderate correlation, respectively, in the functional domain. Allegra et al. (14) investigated the impact of vocal performance on the quality of life of laryngectomized individuals rehabilitated with tracheoesophageal prosthesis using both instruments (V-RQOL and VHI), and they reported significance in the functional scale in both instruments. Both instruments include similar items, and most of the shared items correspond to the functional domain. Another study showed that only two questions do not apply to this particular population. The authors suggested adjusting the scores to improve the instrument’s sensitivity (12). Studies analysing other voice disorders also found a similar correlation between the V-RQOL and VHI (59,60)**.** These findings reinforce the idea that both instruments can be used without the need for a specific protocol on head and neck cancer when identifying voice-related issues.

The correlation found between the VHI and the EORTC QLQ-C30 functional domain in both groups suggests that lower voice handicaps result in better functioning of the patient. The correlation detected between the VHI and EORTC QLQ-H&N35 in the SG group might be accounted for by the fact that the latter instrument includes items on ongoing voice disorders and seeks to establish how much they might interfere with social relationships. Another study also found this same correlation among oesophageal, tracheoesophageal or electrolarynx speakers and non-rehabilitated individuals (54).

Antin et al. (15) investigated the quality of life through EORTC QLQ-C30, EORTC QLQ-H&N35 and VHI instruments in individuals rehabilitated by tracheoesophageal prosthesis, oesophageal voice and electronic larynx, and they highlighted the importance of functional rehabilitation for better quality of life rates.

Other studies found better outcomes for the quality of life functional domains among patients who underwent a total laryngectomy who acquired oesophageal speech (9,13,32).

The correlation found between the EORTC QLQ-C30 functional domain and all other instruments, except the VAS, in both groups indicates that the EORTC QLQ-C30 items address the difficulties met by this population of patients. In addition, this correlation points to the relevance of functional aspects in the lives of patients, and the results in this regard were better for the SG group.

The correlation detected between the global health/quality of life and functional domains of the EORTC QLQ-C30 and EORTC QLQ-H&N35 indicates that fewer symptoms result in better quality of life. Relative to the symptom domain, fewer symptoms in the EORTC QLQ-C30 result in lower numbers on the specific instrument, indicating that these instruments complement each other. EORTC QLQ-H&N35 has been used worldwide with high acceptance by researchers, and most of its scales have good to very good consistency coefficients (61).

The EORTC QLQ-C30 is applied when the aim of a study is to compare different groups of patients, intragroup changes over time or intergroup changes over time. For this reason, scores are calculated for the various scales instead of one global score (62).

The correlation between the FACT-H&N and the EORTC QLQ-C30 global health/quality of life and functional domains and between the EORTC-H&N35 and UW-QOL in the SG group might have occurred because these instruments consider social and family aspects. The correlations suggest that the items in these three instruments are adequate for this population because several studies have emphasized the relevance of social aspects in rehabilitation following laryngectomy (10,52,57).

The correlation found between the UW-QOL and the remainder of the instruments in the SG group and with most instruments in the NSG group shows that the UW-QOL items address physical and functional aspects and are useful for this population of patients. Other studies have found that the UW-QOL is adequate for patients who underwent total laryngectomy and the Brazilian population of patients with head and neck cancer (18,46,63).

The findings of this study showed that the functional domain of individuals from both groups (speakers and non-speakers) was the one that interfered most in the quality of life.

Because the functional scale refers to issues (such as communication problems) that may interfere with the way they are understood due to new voice and ways of performing their activities inside and outside the home (such as leisure, work and daily life activities), the professionals involved in the rehabilitation of these individuals need to pay more attention to this domain to promote gains in quality of life. Among the protocols studied, the EORTC QLQ-C30, EORTC QLQ-H&N35 and UW-QOL protocols were the most relevant to meet the needs of this population in both groups, and one of them could be chosen to assess the quality of life.

Both groups showed a benefit from the highlighted instruments. However, it was not the objective of our study to show which of the two groups presented the best quality of life when comparing the instruments. However, a previous study demonstrated individuals not rehabilitated by alaryngeal methods have a greater commitment to quality of life than those rehabilitated (32).

Among the limitations of the present study is studying a patient population from a single institution. In addition, the sample size was small but within the minimum limit estimated by the sample calculation.

Future multicentre studies can overcome these failures and verify the results for the Brazilian population and for speakers of other languages in addition to further detailing the subscales. New research using these instruments presenting greater correlations in different populations will also be considered for the continuity of this study.

### Conclusion

The findings of the present study suggest that the EORTC QLQ-C30, EORTC QLQ-H&N35 and UW-QOL meet the needs of the target population. The results also indicate that there is no need to use a specific questionnaire for voice because the aforementioned instruments address voice aspects in a manner sufficient for their assessment.

## AUTHOR CONTRIBUTIONS

Raquel ACS contributed in conception and methodological design, data collected, data analyses and interpretation and preparation of the material for publication. Buzaneli EP and Lima HS contributed in data collected. Simões-Zenari M and Kulcsar MAV and Nemr K contributed in conception and design of study, analyses and interpretation of data and reviewed the entire publication submitted. Kowalski LP contributed in reviewed the entire publication submitted.

## Figures and Tables

**Table 1:** Description of the sample in relation to gender. type of surgery. neck dissection (ND). radiotherapy (RT). chemotherapy (CHEMO). tobacco and alcohol consumption.

		GROUP			
		SG		NSG	
		N	%	N	%
Gender	Female	2	10.5	0	0
	Male	17	89.5	19	100.0
Laryngectomy
	TL	15	78.9	18	94.7
	TL+partial ph.	4	21.1	1	5.3
Neck dissection	No information	0	.0	1	5.3
	No	3	15.8	1	5.3
	Yes	16	84.2	17	89.5
RT	No	4	21.1	5	26.3
	Yes	15	78.9	14	73.7
CHEMO	No	13	68.4	12	63.2
	Yes	6	31.6	7	36.8
Tobacco history	No	0	.0	1	5.3
	Yes	19	100.0	18	94.7
Alcohol history	No	3	15.8	3	15.8
	Yes	16	84.2	16	84.2

TL+partial ph= total laryngectomy and partial pharyngectomy; TL= total laryngectomy.

**Table 2:** Description of the sample in relation to age. pos operative and rehabilitation speech.

		GROUP		Total
		SG	NSG	
AGE	Mean	59.6	62.0	60.8
	Median	63.0	63.0	63.0
	Minimum	30	47	30
	Maximum	68	77	77
	P-value	9.0	7.8	8.4
	N	19	19	38
SURGERY (MONTHS)	Mean	27.6	14.5	21.2
	Median	20.6	15.2	18.0
	Minimum	7.0	2.6	2.6
	Maximum	103.5	32.1	103.5
	P-value	22.9	8.8	18.5
	N	19	18	37
SPEECH THERAPY SESSIONS	Mean	25.7	14.5	20.1
	Median	17.0	11.0	15.5
	Minimum	6	3	3
	Maximum	55	31	55
	P-value	16.4	8.6	14.1
	N	19	19	38
